# CYP2E1 overexpression protects COS-7 cancer cells against ferroptosis

**DOI:** 10.21203/rs.3.rs-2702878/v1

**Published:** 2023-03-22

**Authors:** Andres A. Caro, Daniel Barrett, Cristobal Garcia, Weston Northington, Jamya Pinkney, Rayan Shuja, Hannah Stovall

**Affiliations:** Hendrix College; Hendrix College; Hendrix College; Hendrix College; Hendrix College; Hendrix College; Hendrix College

**Keywords:** Ferroptosis, CYP2E1, lipid peroxidation, glutathione, cell death

## Abstract

Ferroptosis is a recently described form of regulated cell death initiated by the iron-mediated one-electron reduction of lipid hydroperoxides (LOOH). Cytochrome P450 2E1 (CYP2E1) induction, a consequence of genetic polymorphisms or/and gene induction by xenobiotics, may promote ferroptosis by contributing to the cellular pool of LOOH. However, CYP2E1 induction also increases the transcription of anti-ferroptotic genes that regulate the activity of glutathione peroxidase 4 (GPX4), the main ferroptosis inhibitor. Based on the above, we hypothesize that the impact of CYP2E1 induction on ferroptosis depends on the balance between pro- and anti-ferroptotic pathways triggered by CYP2E1. To test our hypothesis, ferroptosis was induced with class 2 inducers (RSL-3 or ML-162) in mammalian COS-7 cancer cells that don’t express CYP2E1 (Mock cells), and in cells engineered to express human CYP2E1 (WT cells), and the impact on viability, lipid peroxidation and GPX4 was assessed. CYP2E1 overexpression protected COS-7 cancer cells against ferroptosis, evidenced by an increase in the IC_50_ and a decrease in lipid ROS in WT versus Mock cells after exposure to class 2 inducers. CYP2E1 overexpression produced an 80% increase in the levels of the GPX4 substrate glutathione (GSH). Increasing GSH in Mock cells protected cells against ferroptosis by ML-162. Depleting GSH, or inhibiting Nrf2 in WT cells reverted the protective effect mediated by CYP2E1, causing a decrease in the IC_50_ and an increase in lipid ROS after exposure to ML-162. These results show that CYP2E1 overexpression protects COS-7 cancer cells against ferroptosis, an effect probably mediated by Nrf2-dependent GSH induction.

## Introduction

**Figure 1:**
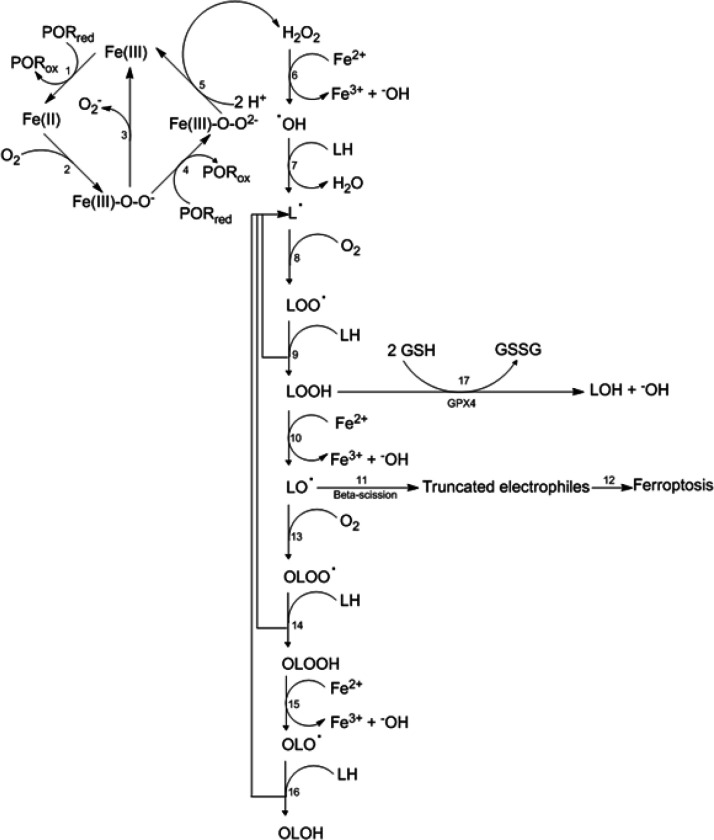
Proposed reaction sequence from CYP2E1 leading to ferroptosis. CYP2E1 produces ROS (primarily O_2_^−^and H_2_O_2_) during its uncoupled catalytic cycle (upper left). This uncoupled cycle starts with the one-electron reduction of the ferric heme iron in CYP2E1 (Fe(III)) to Fe(II) by NADPH-reduced cytochrome P450 oxidoreductase (POR_red_) (1). The oxygenation of Fe (II) produces the ferric-superoxide complex Fe (III)-O-O^−^ (2). This intermediate can revert back to Fe (III) releasing O_2_^−^ (3), which further dismutates to H_2_O_2_ (not shown). Alternatively, one-electron reduction of Fe (III)-O-O^−^ by POR_red_ produces the peroxy-ferric intermediate Fe (III)-O-O^2−^(4) that after protonation reverts back to H_2_O_2_ and the original Fe(III) (5). Autoxidation (upper right), i.e. the non-enzymatic reaction of molecular oxygen (O_2_) with polyunsaturated fatty acids (LH), produces lipid hydroperoxides (LOOH) as primary products. Its mechanism involves the initial oxidation of LH to L^●^ with a hydroxyl radical species (^●^OH) (7) produced by the reaction of H_2_O_2_ with ferrous iron (Fe^2+^) (6), and is followed by the oxygenation of L^●^ to LOO^●^ (8), and the propagation of the chain reaction by the oxidation of LH back to L^●^ by LOO^●^ (9). Ferroptosis is initiated by the oneelectron reduction of the resulting lipid hydroperoxides (LOOH) by Fe^2+^ (10), which produces alkoxyl radical (LO^●^) that in turn generates truncated electrophiles by beta- scission (11). These truncated electrophiles react with specific nucleophilic targets in proteins, executing ferroptosis (12). Oxygenation of LO^●^ (13), followed by oxidation of LH by the resulting epoxyallylic hydroperoxyl OLOO^●^ radical (14) propagates ferroptosis. Epoxyallylic alkoxyl radical (OLO^●^) generated by ferrous iron-mediated reduction of epoxyallylic hydroperoxides (OLOOH) (15) further propagates ferroptosis (16). Ferroptosis is limited by the activity of the enzyme GPX4, which reduces LOOH to LOH using GSH as substrate (17). See text for details.

Ferroptosis is a recently described form of regulated cell death initiated by the iron-mediated one-electron reduction of lipid hydroperoxides (LOOH) to lipid alkoxyl radicals (LO^●^), and propagated by rounds of peroxyl and alkoxyl radical- mediated polyunsaturated fatty acid (LH) oxidation (Fig. 1) (Girotti 2008; Maiorino et al. 2018; Sha et al. 2018; Miotto et al. 2020). Truncated electrophiles derived from the beta-scission of LO^●^ are proposed as executioners of ferroptosis, by readily interacting with nucleophilic protein targets (Fig. 1) (Rezende et al. 2022; Stoyanosky et al. 2019).

Lipid hydroperoxides (LOOH) are generated from LH and O_2_ by two primary mechanisms: a non-enzymatic, hydrogen peroxide (H_2_O_2_)- and ferrous iron (Fe^2+^)- dependent free radical chain reaction called autoxidation (Fig. 1) (Girotti 1998; Schafer et al. 2000), and an enzyme-catalyzed process mediated by lipoxygenases (LOXs), cyclooxygenases (COXs), or cytochrome P450 oxidoreductases (PORs) coupled mainly to cytochrome P450 monooxygenases (P450s) (Foret et al. 2020). Any process that contributes to the cellular pool of LOOH could increase the rate of initiation of lipid peroxidation and could sensitize cells to ferroptosis (Sha et al. 2018).

Cells have evolved at least three defense mechanisms to suppress ferroptosis: a) glutathione peroxidase-4 (GPX4), which uses glutathione (GSH) to reduce and detoxify LOOH (Fig. 1); b) ferroptosis suppressor protein 1 (FSP1), which reduces ubiquinone to ubiquinol on the plasma membrane, which further traps and inactivates lipid peroxyl and alkoxyl radicals, and c) dihydroorotate dehydrogenase (DHODH), which carries out a similar reaction to that of FSP1 but on the inner mitochondrial membrane (Mao et al. 2021). Increased activity of gene products that decrease the overall pool of LOOH, such as GPX4, γ-glutamylcysteine synthetase (γGCS, the rate-limiting enzyme for the synthesis of the GPX4 substrate GSH), and SLC7A11 (also known as xCT, a transporter that promotes cystine uptake and GSH biosynthesis) could decrease the rate of initiation of lipid peroxidation and could suppress ferroptosis (Sha et al. 2018; Koppula et al. 2018).

Several lines of evidence suggest that PORs are major contributors to ferroptosis initiation. For example, in several cancer cell lines susceptible to ferroptosis and lacking significant expression of LOXs, genetic POR depletion prevented ferroptosis triggered by class 1 (xCT inhibitors) and class 2 (GPX4 inhibitors) inducers, probably by preventing the cycling between Fe(II) and Fe(III) in the heme component of P450s (Zou et al. 2020). In steroidogenic adrenocortical (but not in non-P450 expressing) cells, ketoconazole (a nonselective P450 inhibitor) prevented ferroptosis induced by GPX4 inhibition, probably by preventing uncoupled P450 activity (Weigand et al. 2020). Knockdown of POR in HeLa cells resulted in resistance to ferroptosis triggered by class 1 and 2 inducers (Yan et al. 2021). However, preventing the interaction of POR with P450 did not prevent ferroptosis in this case, suggesting that other electron acceptors from POR (such as cytochrome b5, heme oxygenase, or squalene monooxygenase), or the NADPH oxidase activity of POR *per se* could also be responsible for ferroptosis initiation (Yan et al. 2021). The POR/P450 system produces LOOHs as primary oxidation products of polyunsaturated fatty acids (LH) via reactive oxygen species (ROS, including O_2_^−^, H_2_O_2_ and ^●^OH)-mediated autoxidation (Caro and Cederbaum 2006; Yan et al. 2021).

The 2E1 isoform (CYP2E1) of P450 is among the most active in the generation of ROS. CYP2E1-dependent ROS are generated during its catalytic cycle after two single-electron transfer reactions from NADPH catalyzed by POR. ROS generation occurs either by the decomposition of the ferric-superoxide complex that produces superoxide anion (O_2_^−^, which further dismutates to H_2_O_2_), or the decomposition of the peroxy-ferric intermediate that produces H_2_O_2_ (Fig. 1) (Caro and Cederbaum 2004). This suggests that CYP2E1 induction (a consequence of genetic polymorphisms or/and gene induction by xenobiotics) may promote ferroptosis by contributing to the cellular pool of LOOH via ROS-mediated autoxidation of LH (Fig. 1). However, CYP2E1 induction also increases the transcription (in an ROS-dependent manner) of γGCS (Mari and Cederbaum 2000), GPX4 (Oliva et al. 2011) and xCT (Choi et al. 2019), potentially contributing to ferroptosis suppression by activating GPX4 and limiting the pool of LOOH (Fig. 1). Based on the above, we hypothesize that the impact of CYP2E1 induction on ferroptosis depends on the balance between pro- and anti-ferroptotic pathways triggered by POR/CYP2E1.

To test our hypothesis, ferroptosis was induced with class 2 inducers (RSL-3 or ML-162) in mammalian COS-7 cancer cells that don’t express CYP2E1, and in cells engineered to express human CYP2E1, and the impact on viability, lipid peroxidation, GPX4, and GSH levels was assessed.

## Materials And Methods

### Chemicals:

Fetal bovine serum (FBS) was from Neuromics (Edina, MN). Ferroptosis inducers and inhibitors were from Cayman Chemical (Ann Arbor, MI). BODIPY^™^ 581/591 C11 (C11BODIPY) was from ThermoFisher Scientific (Waltham, MA). Most of the other chemicals used were from Sigma-Aldrich (St. Louis, MO).

### Cell lines:

COS-7 cells transduced with the empty retroviral pBABE-puro vector (Mock cells), or with the retroviral pBABE-puro vector containing wild-type CYP2E1 cDNA (WT cells) or a mutated CYP2E1 construct (W23/30R cells) were maintained in Dulbecco’s modified Eagle’s medium (DMEM) with 25 mM glucose, supplemented with 10% FBS (v/v) in the presence of added 1% penicillin/streptomycin in a 5% CO_2_:95% air (v/v) incubator at 37°C (Bansal et al. 2013). Puromycin (5 μg/ml) was added after every four passages to maintain the integrity of the cells expressing CYP2E1. Cells were sub-cultured at a 1:5 ratio once a week. For the experiments, cells were plated at a density of 25,000 cells/ml in DMEM supplemented with 25 mM glucose, 5% FBS (v/v) and 1% penicillin/streptomycin. Cells were treated for 24h as described under Results in a 5% CO_2_:95% air (v/v) incubator at 37°C, followed by the biochemical assays described below. The cells were a gift from Dr. Narayan Avadhani, University of Pennsylvania School of Veterinary Medicine, Philadelphia, PA.

### Viability:

Cell viability was determined by the 3-(4,5-dimethylthiazol-2-yl)-2,5-diphenyl-2H-tetrazolium bromide (MTT) assay (Carmichael et al. 1987). Brie y, after the treatment period, the medium was aspirated, and replaced with fresh medium without FBS containing MTT at 1 mg/mL. Plates were incubated at 37°C in a humidified 5% CO_2_ atmosphere for 1h. At the end of the incubation period, the medium was aspirated, and the formazan product was dissolved in 0.3 mL of n-propanol. Cell viability was evaluated by measuring the absorbance at the test wavelength of 570 nm, correcting it by the absorbance at the reference wavelength of 630nm, and converting this value to percentage of the control (defined as cells without treatment). Cytotoxic effects were expressed as IC_50_ (the concentration of drug that reduces viability by 50% of the non-treated control). The IC_50_ was determined using the four-parameter logistic function y = D + (A-D)/1 + 10(x-logC) B, with parameter C representing the estimation of IC_50_, using the Sigma Plot 12.0 software program from Systat Software (Richmond, CA).

### Lipid peroxidation:

Lipid ROS were determined by the level of oxidation of the polyunsaturated butadienyl portion of C11BODIPY (Martinez et al. 2020). After the treatments, the medium was removed and replaced with 0% FBS- DMEM containing 2.5 μM C11BODIPY. After a 20-minute incubation at 37°C and 5% CO_2_, cells were washed with PBS, trypsinized, resuspended in 0% FBS- DMEM, and analyzed by flow cytometry using an Accuri C6 Flow Cytometer (BD Biosciences, San Jose, CA, USA) equipped with a 488 nm, 50 mW solid-state laser. C11BODIPY oxidation results in a shift of the fluorescence emission peak from ~ 590nm to ~ 510nm. C11BODIPY oxidation was reported as the percentage of cells that exhibit high fluorescence emission intensity in the FL1 fluorescence channel (530 ± 30 nm).

### Glutathione levels:

After the treatments, cells were trypsinized, resuspended in PBS, and counted with a hemacytometer. Up to 8000 cells were dispensed at 50μl per well into 96-well chemiluminescence plates. Glutathione levels were measured using the GSH-Glo glutathione luminescent assay as per manufacturer’s instructions (Promega, Madison, WI). Chemiluminescence was measured using a Synergy 2 multi-function microplate reader from BioTek (Winooski, VT), and normalized to the vehicle control. GSH detection by chemiluminescence was linear with cell density up to 8,000 cells/well in both Mock and WT cells (data not shown).

### CYP2E1 activity:

CYP2E1 activity in intact cells (*in situ*) or in CYP2E1 baculosomes (microsomes prepared from insect cells infected with recombinant baculovirus containing human CYP2E1 and POR, *in vitro*) was determined as the O-demethylation of 7 -methoxy-4-tri uoromethylcoumarin (7-MFC) to 7-hydroxy-4-tri uoromethylcoumarin (7-HFC). For the *in situ* assay, 1 × 10^6^ Mock or WT cells were plated in 1 mL phosphate buffered saline (PBS) supplemented with 5.5 mM glucose, 1 mM CaCl_2_ and 1.8 mM MgCl_2_, at 37°C in a 5% CO_2_ incubator. Reactions were initiated by the addition of 7-MFC in acetonitrile at 10 μM. After the indicated time intervals, cells were scraped and fluorescence of the suspension was determined at 410/510 nm excitation/emission using a Hitachi Model F-4500 fluorimeter (Caro and Cederbaum 2007). For the *in vitro* assays, the reaction system contained 100 mM Tris-HCl buffer (pH 7.4), 5 mM MgCl_2_, 10 μM 7-MFC and CYP2E1 baculosomes at 0.25 mg protein/mL. The reaction was initiated by the addition of NADPH at 0.5 mM, and terminated at 30 min of incubation by the addition of 80% acetonitrile/20% 0.5 M Tris Base (Martikainen et al. 2012). Fluorescence was determined at 360 ± 40/528 ± 20 nm using a Synergy 2 multi-function microplate reader from BioTek (Winooski, VT). O-demethylation of 7-MFC in vitro by human CYP2E1 baculosomes was linear with respect to incubation time (up to 60 min), and protein concentration (up to 0.5 mg/mL) (data not shown).

### GPX4 activity:

GPX4 activity in cell extracts was measured by recording the NADPH oxidation rate at 340 nm in the presence of GSH, GSSG reductase, and phosphatidylcholine hydroperoxide (PCOOH) (Stolwijk et al. 2020). Brie y, the reaction mixture contained 0.1 M Tris-HC1 (pH 7.4), 2 mM EDTA, 0.1% (v/v) Triton X-100, 0.2 mM NADPH, 3 mM GSH, 1.5 U/mL of GSH reductase, and the cell extract containing enzyme activity. After recording the baseline rate of nonspecific NADPH oxidation (which was subtracted from the activity), the reaction was started by adding 10 μM PCOOH. The increase in the rate of oxidation of NAPDH upon addition of PCOOH is considered to be GPX4-specific (Stolwijk et al. 2020). Phosphatidylcholine hydroperoxide was prepared as described in (Stamenkovic et al. 2021).

### Statistics:

Data are expressed as means ± S.E. of the mean from 3–5 independent experiments. One-way analysis of variance with subsequent post hoc comparisons by Student-Newman-Keuls was performed. p < 0.05 was considered as statistically significant.

## Results

### 1) CYP2E1 overexpression in COS-7 cells limits cell death triggered by GPX4 inhibition.

**Figure 2:**
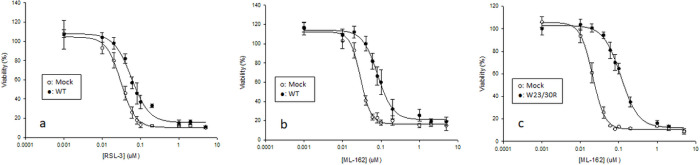
CYP2E1-overexpressing Cos-7 cells are less sensitive to class II ferroptosis-inducing agents than non-CYP2E1 expressing Cos-7 cells. (a) MTT viability assay results after treating mock-transfected (Mock, o) or human-CYP2E1-transfected (WT, ●) COS-7 cells with increasing concentrations of RSL3 (from 0.001 to 8 μM) for 24 h. (b) MTT viability assay results after treating mock-transfected (Mock, o) or human-CYP2E1-transfected (WT, ●) COS-7 cells with increasing concentrations of ML-162 (from 0.001 to 8 μM) for 24 h. (c) MTT viability assay results after treating mock-transfected (Mock, o) or mt-CYP2E1-transfected (W23R/30R, ●) COS-7 cells with increasing concentrations of ML-162 (from 0.001 to 8 μM) for 24 h.

Cell death was experimentally induced by irreversible inhibition of GPX4 with Ras-selective lethal small molecule 3 (RSL-3) via its reactive chloroacetamide moiety. The IC_50_ of RSL-3 was higher in WT cells (COS-7 cells overexpressing human wild type CYP2E1) (0.082 μM) than in Mock cells (COS-7 cells not expressing any cytochrome P450) (0.039 μM) (Fig. 2a). ML-162 (a structurally different chloroacetamide GPX4 inhibitor) was also used in parallel experiments. ML-162 was used to verify the GPX4-dependence of RSL-3-mediated effects (Stockwell and Jiang 2020): the probability that similar results observed with RSL-3 and ML-162 are not caused by chloroacetamide-mediated GPX4 inhibition, but by an off-target effect, is minimal due to their significant structural dissimilarities. The IC_50_ of ML-162 was higher in WT cells expressing human wild type CYP2E1 (0.089 μM) than in Mock cells not expressing any cytochrome P450 (0.035 μM) (Fig. 2b). In either cell type, the IC50 of RSL-3 was not significantly different to that of ML-162. Importantly, stable transfection of the same cell line with mutated CYP2E1 targeted to mitochondria (W23/30R cells) also increased the IC_50_ of RSL-3 with respect to Mock cells (from 0.040 to 0.15 μM) (Fig. 2c), validating that CYP2E1 irrespective of its localization, and not an artifact of clonal selection, is responsible for the increased IC_50_ of GPX4 inhibitors in WT cells.

### 2) GPX4 inhibition induces ferroptosis in both CYP2E1-expressing and non-expressing COS-7 cells.

**Figure 3:**
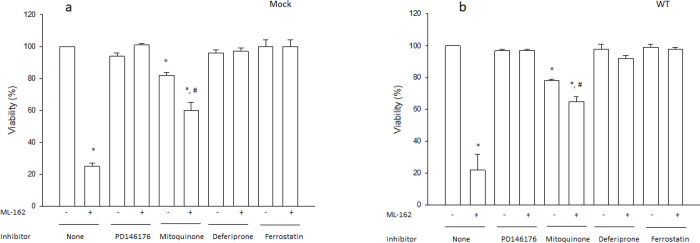
ML-162 induces ferroptosis in both CYP2E1- expressing and non-expressing Cos-7 cells. Mock-transfected (Mock, a) or human-CYP2E1-transfected (WT, b) COS-7 cells were exposed to the ferroptosis inducer ML-162 at 0 uM (ML-162 -) or at a concentration that induced 75% cell death according with data from Fig. 2 (ML-162 +). The inhibitors (PD146176, mitoquinone, deferiprone and ferrostatin) were added at the same time as ML-162 at the optimal concentrations reported by Soriano-Castell et al. (2021) (5, 1, 100 and 10 uM, respectively). After a 24h incubation, viability was assessed as described under Materials and Methods. * Significantly different (p< 0.05, ANOVA) compared with control cells without any addition. # Significantly different (p< 0.05, ANOVA) compared with cells incubated with the same inhibitor but without ML-162.

**Figure 4:**
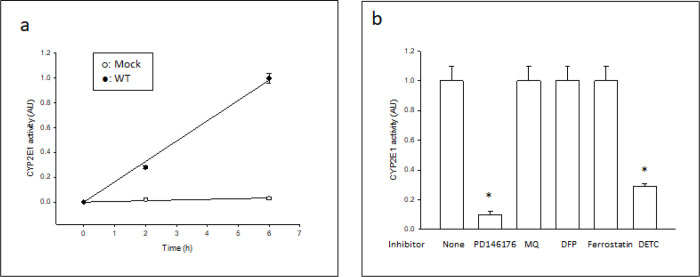
Effect of ferroptosis inhibitors on CYP2E1 activity. (a) Human CYP2E1-transfected Cos-7 cells actively O-demethylate 7-MFC. Mock (o) or WT (●) cells were incubated for variable periods in the presence of 5 uM 7-MFC. The fluorescence of the O-demethylation product 7-HFC in the cellular suspension (expressed in arbitrary units AU) was then determined at 409/530 nm. (b) O-demethylation of 7-MFC was determined in human CYP2E1-expressing microsomes exposed to 5 uM 7-MFC and ferroptosis inhibitors PD146176, MQ (mitoquinone), DFP (deferiprone) and ferrostatin at the concentrations used in the cellular assays, or a specific CYP2E1 inhibitor (DETC, diethyldithiocarbamate) at 5 uM. CYP2E1 activity was expressed as relative to the untreated control (None). * Significantly different (p< 0.05, ANOVA) compared with control microsomes without any addition.

To confirm that GPX4 inhibition induced ferroptosis in both Mock and WT cells, a pharmacological fingerprint based on inhibitors that can discriminate between ferroptosis and other forms of cell death was used (Soriano-Castell et al. 2021). For this analysis, we selected (based on the results shown in Fig. 2b) doses of ML-162 that decreased viability by 75% in Mock or WT cells, in order to achieve a similar level of cell death (higher than 50% but lower than 100%) in both cell types. Cell death induced by ML-162 in both Mock (Fig. 3a) and WT (Fig. 3b) cells was significantly prevented by PD146176 (a 15-LOX inhibitor), mitoquinone (a mitochondrially-targeted antioxidant), deferiprone (an iron chelator) and ferrostatin (a lipid peroxidation inhibitor), a fingerprint that discriminates ferroptosis from other types of cell death (Soriano-Castell et al. 2021). To rule out CYP2E1 inhibition as a possible off-target effect of the inhibitors listed above, the effect of these inhibitors on CYP2E1 activity was evaluated. WT cells (but not Mock cells) expressed active human CYP2E1 as determined by O-demethylation of 7-MFC *in situ* (Fig. 4a). O-demethylation of 7-MFC *in vitro* by human CYP2E1 baculosomes was significantly inhibited by diethyldithiocarbamate (a specific CYP2E1 inhibitor), but not by the ferroptosis inhibitors mitoquinone, deferiprone, or ferrostatin, at the concentrations used in the cellular assays (Fig. 4b). PD146176, however, at the concentration used in the cellular assays, significantly inhibited CYP2E1 activity *in vitro* (Fig. 4b), suggesting that the inhibition by PD146176 of ML-162-induced ferroptosis in WT cells could be partially mediated by CYP2E1 inhibition and not only by 15-LOX inhibition. The ferroptosis inhibitors tested did not affect the fluorescence of 7-HFC at the concentrations used in the cellular assays (data not shown).

### 3) CYP2E1 overexpression in COS-7 cells prevents the early increase in lipid ROS caused by GPX4 inhibition.

**Figure 5:**
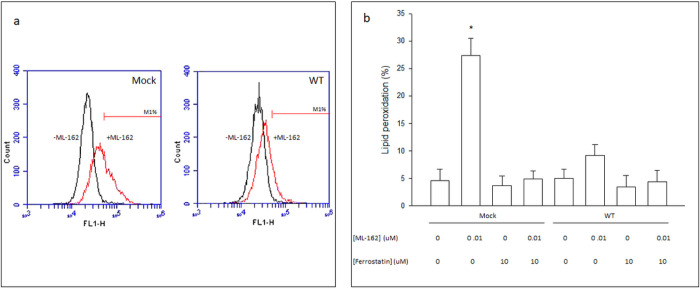
Lipid ROS levels are lower in CYP2E1-expressing than in non-CYP2E1 expressing Cos-7 cells after exposure to non-lethal concentrations of ML-162. (a) Mock (left panel) or WT (right panel) Cos-7 cells were treated with 0 (−ML-162) or 0.01 uM (+ML-162) ML-162 for 24 h and harvested to determine lipid ROS levels by C11-BODIPY staining and flow cytometry analysis. M1% represents the percentage of cells in the population that have high FL1 fluorescence. (b) Mock or WT Cos-7 cells were treated for 24 h with 0 or 0.01 uM ML-162, in the presence or absence of 10 uM ferrostatin, and harvested to determine lipid ROS levels by C11-BODIPY staining and flow cytometry analysis. Lipid peroxidation is expressed as % of cells with high FL1 fluorescence (M1%). * Significantly different (p< 0.05, ANOVA) compared with control Mock cells without any addition.

The intracellular oxidation of C11BODIPY is mainly caused by the oxygen-centered radicals (i.e. lipid peroxyl, alkoxyl) produced during lipid peroxidation (Michel et al. 2020). C11BODIPY oxidation is therefore an indirect manifestation of the lipid ROS ux, associated with the initiation and propagation of the lipid peroxidation that characterizes ferroptosis (Wiernicki et al. 2020). Therefore, an increase in C11BODIPY oxidation should be an early event (i.e. occurring under conditions prior to overt cell death) in the ferroptotic process. To evaluate lipid ROS ux, Mock and WT cells were treated with 0.01 μM ML-162 for 24h (prior to overt cell death), followed by the analysis of C11BODIPY oxidation. In Mock cells, but not in WT cells, ML-162 produced a significant 6-fold increase in the percentage of cells exhibiting high FL1 fluorescence (M1%) with respect to untreated cells (Fig. 5a and b). This increase was completely prevented by ferrostatin, confirming the specificity of the assay (Fig. 5b). No significant differences were observed in C11BODIPY oxidation between untreated Mock and WT cells (Fig. 5b).

### 4) Increased glutathione in CYP2E1-overexpressing COS-7 cells protects against ferroptosis.

**Figure 6:**
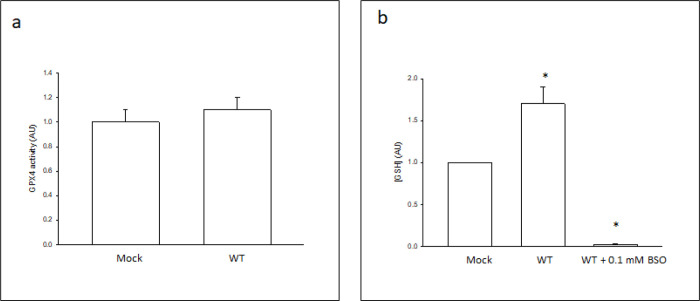
GPX4 activity and GSH levels in Cos-7 cells. (a) GPX4 activity in mock-transfected (Mock) or human CYP2E1-transfected (WT) Cos-7 cells was determined as described under Materials and Methods, and expressed relative to the Mock control. (b) GSH levels in mock-transfected (Mock) Cos-7 cells, human CYP2E1-transfected (WT) Cos-7 cells, or WT cells incubated for 24 h with 0.1 mM BSO (WT + 0.1 mM BSO) was determined as described under Materials and Methods, and expressed relative to the Mock control. * Significantly different (p< 0.05, ANOVA) compared with control Mock cells without any addition.

**Figure 7:**
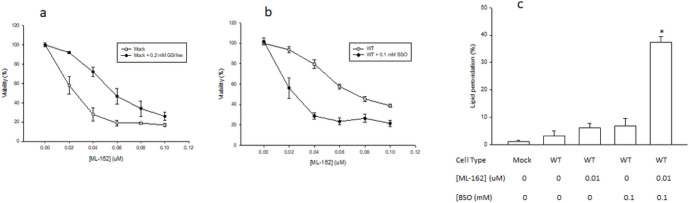
Effect of the manipulation of cellular GSH levels on ferroptosis. (a) MTT viability assay results in mock-transfected cells treated for 24 h with solvent control (Mock) or 0.2 mM GSHee (Mock + 0.2 mM GSHee), and increasing concentrations of ML-162 (from 0 to 0.1 uM). (b) MTT viability assay results in human-CYP2E1-transfected cells treated for 24 h with solvent control (WT) or 0.1 mM BSO (WT + 0.1 mM BSO), and increasing concentrations of ML-162 (from 0 to 0.1 uM). (c) Mock or WT Cos-7 cells were treated for 24 h with 0 or 0.01 uM ML-162, in the presence or absence of 0.1 mM BSO, and harvested to determine lipid ROS levels by C11-BODIPY staining and flow cytometry analysis. Lipid peroxidation is expressed as % of cells with high FL1 fluorescence (M1%). * Significantly different (p< 0.05, ANOVA) compared with control Mock cells without any addition.

Because GPX4 is the master regulator of ferroptosis due to its unique ability to directly reduce phospholipid hydroperoxides in membranes and prevent lipid peroxidation (Chen et al. 2021), we evaluated the possibility that CYP2E1 prevents the lipid peroxidation and ferroptosis induced by ML-162 by increasing GPX4 activity (phospholipid hydroperoxide reduction) or GPX4 substrate (GSH) levels. There were no significant differences in the activity of GPX4 between Mock and WT cells (Fig. 6a). A significant 1.8-fold increase in GSH levels was detected in WT cells with respect to Mock cells (Fig. 6b). An inhibitor of γGCS, buthionine sulfoximine (BSO), decreased GSH levels by more than 95% in WT cells, confirming the specificity of the assay (Fig. 6b). If the increased GSH levels in CYP2E1-overexpressing cells protects against ferroptosis, then: i) increasing GSH levels in Mock cells should prevent ferroptosis, and ii) decreasing GSH levels in WT cells promote ferroptosis. To test these possibilities, we increased GSH levels in Mock cells with the use of a cell-permeable form of GSH, glutathione ethyl ester (GSHee), and found that the IC_50_ of ML-162 increased from 0.020 μM in the absence of GSHee to 0.052 μM in the presence of 0.2 mM GSHee (Fig. 7a). In addition, we decreased GSH levels in WT cells with the use of BSO, and found that the IC_50_ of ML-162 decreased from 0.104 μM in the absence of BSO to 0.020 μM in the presence of 0.1 mM BSO (Fig. 7b). Because an increase in lipid ROS is an early event during ferroptosis (Wiernicki et al. 2020) we evaluated the effect of GSH depletion on lipid ROS levels in WT cells. C11BODIPY oxidation (expressed as percentage of cells exhibiting high C11BODIPY FL1 fluorescence) in WT cells exposed to a non-lethal concentration of ML-162 was 6.7 ± 0.5% in the absence of BSO, a value that was not significantly different from that of untreated Mock or WT cells, or WT cells exposed to 0.1 mM BSO (Fig. 7c). C11BODIPY oxidation increased to 36.0 ± 4.0% in WT cells exposed to a non-lethal concentration of ML-162 in the presence of 0.1 mM BSO, a significant 5.4-fold increase (Fig. 7c).

### 5) CYP2E1-induced protection from ferroptosis in COS-7 cells is prevented by Nrf2 inhibition.

**Figure 8:**
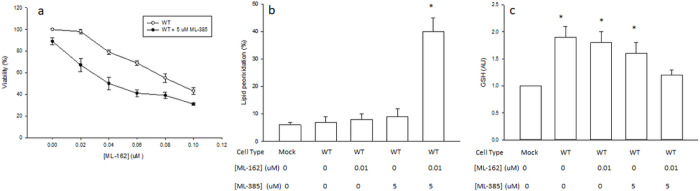
Nrf2 inhibition in CYP2E1-overexpressing Cos-7 cells promoted ferroptosis and GSH depletion. (a) MTT viability assay results in human-CYP2E1-transfected cells treated for 24 h with solvent control (WT) or 5 uM ML-385 (WT + 5 uM ML-385), and increasing concentrations of ML-162 (from 0 to 0.1 uM). (b) Mock or WT Cos-7 cells were treated for 24 h with 0 or 0.01 uM ML-162, in the presence or absence of 5 uM ML-385, and harvested to determine lipid ROS levels by C11-BODIPY staining and flow cytometry analysis. Lipid peroxidation is expressed as % of cells with high FL1 uorescence (M1%). (c) Mock or WT Cos-7 cells were treated for 24 h with 0 or 0.01 uM ML-162, in the presence or absence of 5 uM ML-385. GSH levels were then were determined as described under Materials and Methods, and expressed relative to the Mock control. * Significantly different (p< 0.05, ANOVA) compared with control Mock cells without any addition.

Nrf2 plays a key role in the adaptive response against increased oxidative stress caused by CYP2E1 in cultured cells, through up-regulation of γGCS and increase in GSH levels (Gong and Cederbaum 2006). Therefore, we tested the effect of a specific chemical Nrf2 inhibitor (ML-385) on ferroptosis and GSH levels in WT cells exposed to ML-162. ML-385 by itself did not show significant toxicity in WT cells at the concentration used (Fig. 8a). The IC_50_ of ML-162 in WT cells decreased from 0.088 μM in the absence of ML-385 to 0.035 μM in the presence of 5 μM ML-385 (Fig. 8a). The percentage of cells with high levels of lipid ROS (a pro-ferroptotic condition) increased from 9% in WT cells exposed to a non-lethal concentration of ML-162 to 38% in WT cells exposed to a non-lethal concentration of ML-162 in the presence of 5 uM ML-385, a significant 4-fold increase (Fig. 8b). To test if Nrf2 activity is required for the prevention of ferroptosis in CYP2E1-exprressing cells through the maintenance of GSH levels, we determined GSH levels in cells treated with ML-162 in the presence or absence of the Nrf2 inhibitor ML-385. Nrf2 inhibition decreased GSH levels in WT-cells exposed to ML-162 prior to overt cell death, suggesting that Nrf2 activity is required to maintain GSH levels in CYP2E1-overexpressing cells under ferroptosis conditions (Fig. 8c).

## Discussion

We conclude that CYP2E1 overexpression protects COS-7 cancer cells against ferroptosis based on the following observations: a) the IC_50_ of class 2 inducers (RSL-3 and ML-162) was higher in CYP2E1-overexpressing (WT and W23/30R) than in non-CYP2E1 expressing (Mock) COS-7 cells, and b) ML-162 at 0.01 μM (prior to overt cell death in Mock cells) increased lipid peroxidation (central to the execution of ferroptosis) in Mock but not in WT cells. The mode of cell death by ML-162 was confirmed to be ferroptosis by its pharmacological fingerprint, which includes sensitivity to LOX, lipid peroxidation, iron and mitochondrial oxidant inhibitors. The fact that a specific inhibitor of 15-LOX such as PD146176 prevented ferroptosis by ML-162 in Mock cells suggests that 15-LOX activity mediates LOOH accumulation and ferroptosis in non-CYP2E1 expressing (Mock) Cos-7 cells. PD146176 inhibited ferroptotic cell death in other cell lines exposed to class 2 inducers, also suggesting the involvement of 15-LOX: HT1080 (human brosarcoma) (Shintoku et al. 2017), HEK293 (human embryonic kidney) (Shah et al. 2018), and Jurkat (human T-cell acute lymphoblastic leukemia) (Probst et al. 2017) cells. However, our results show that PD146176 is also a CYP2E1 inhibitor *in vitro*, so through the use of PD146176 alone it is not possible to discard the contribution of CYP2E1 to ferroptosis in CYP2E1-overexpressing (WT) Cos-7 cells. PD146176 was recently identified as a cytochrome P450 epoxygenase inhibitor in human EA.hy926 endothelial cells (Du et al. 2022), so results with PD146176 should be interpreted with caution in cytochrome P450- expressing systems.

Our results suggest that the anti-ferroptotic response in CYP2E1-overexpressing cells is mediated by increased GSH levels. The experimental evidence to support this conclusion is: a) GSH levels were 80% higher in WT than in Mock cells; b) decreasing GSH levels in WT cells by inhibiting γGCS with BSO decreased the IC_50_ of ML-162, blunting the protection induced by CYP2E1; c) exposing Mock cells to a cell- permeable form of glutathione (GSH-ee) increased the IC_50_ of ML-162, indicating decreased toxicity under high GSH levels; d) decreasing GSH levels in WT cells by inhibiting γGCS with BSO increased lipid ROS (a trigger for ferroptosis) after exposing cells to non-lethal doses of ML-162, blunting the protection induced by CYP2E1. GSH levels directly modulates ferroptosis sensitivity in this and other cellular models. For example, GSH depletion promoted ferroptosis in human lens epithelial cells exposed to RSL3 (Wei et al. 2021), in HepG2 human hepatoma cells starved of cystine (Yu and Long 2016), and in PC12 rat pheochromocytoma cells exposed to erastin (Liu et al. 2020). GSH might prevent ferroptosis through enzymatic pathways including the prevention of H_2_O_2_ accumulation (mainly via glutathione peroxidase-1 or GPX1), the prevention of LOOH accumulation (via GPX4), or the detoxi cation of lipid-derived electrophiles (via glutathione S-transferase or GST) (van Kessel et al. 2022).

CYP2E1 upregulates Nrf2 signaling in rat hepatocytes and HepG2 cells in an ROS-dependent manner, which induces the transcriptional activation of the rate limiting-enzyme in GSH synthesis (γGCS), and increases GSH levels (Gong and Cederbaum 2006). Our results suggest that this mechanism could mediate ferroptosis resistance in CYP2E1-overexpressing COS-7 cells: a) ML-385 (a chemical Nrf2 inhibitor) decreased the IC_50_ of ML-162 in WT cells to levels comparable to that in Mock cells, blunting the protection induced by CYP2E1; b) lipid ROS (a trigger for ferroptosis) increased after exposing WT cells to non-lethal doses of ML-162 under Nrf2 inhibition; and c) ML-385 decreased GSH levels prior to cell death in WT cells exposed to ML-162. However, other Nrf2-dependent pathways potentially triggered by CYP2E1 could also in part mediate this increased resistance. For example, Nrf2-dependent microsomal glutathione S-transferase (MGST) expression was induced in CYP2E1-overexpressing HepG2 cells (Mari and Cederbaum 2001), and contributed to ferroptosis resistance in pancreatic cancer cells by inhibiting ALOX5 activity (Kuang et al. 2021). Also, Nrf2-dependent heme oxygenase 1 (HO-1) expression was induced in CYP2E1-overexpressing HepG2 cells (Gong and Cederbaum 2006), and contributed to ferroptosis resistance in hepatocellular carcinoma cells by modifying iron metabolism and lipid peroxidation (Sun et al. 2016). Lastly, xCT expression was induced in CYP2E1-overexpressing primary mouse hepatocytes (Choi et al. 2019), and contributed to ferroptosis resistance in Nrf2- knockdown F98 rat glioma cells (Fan et al. 2017).

Ferroptosis is a natural tumor-suppressor mechanism, and has been proven to be effective in anticancer therapy (Cai et al. 2022; Lei et al. 2022). CYP2E1 induction is linked to the development of chemically-mediated cancers, via ROS or procarcinogen activation (Trafalis et al. 2010). For example, CYP2E1 expression is higher in breast tumors (Kapucuoglu et al. 2003), in the MGC-803 human gastric cancer cell line (Wang et al. 2020), in the esophageal mucosa of patients with esophageal squamous cell carcinoma (Bergheim et al. 2007), and in adjacent nontumor tissues from hepatocellular carcinoma patients (Ho et al. 2004), than in their normal counterparts. Based on the above, CYP2E1 induction could further promote carcinogenesis via inhibiting natural ferroptosis or enhancing resistance to ferroptotic treatments.

In conclusion, although POR-P450 activity has been associated with increased pro-ferroptotic generation of lipid hydroperoxides in other systems, competing anti-ferroptotic pathways shifted the pro-anti ferroptotic balance towards anti-ferroptosis in CYP2E1-overexpressing Cos-7 cells. Nrf2-dependent GSH induction is one factor that could mediate the net anti-ferroptotic effect of CYP2E1 overexpression.

## Data Availability

Data sharing not applicable to this article as no datasets were generated or analyzed during the current study.
